# Alterations in bile acid kinetics after bariatric surgery in patients with obesity with or without type 2 diabetes

**DOI:** 10.1016/j.ebiom.2024.105265

**Published:** 2024-08-02

**Authors:** Annika Wahlström, Ömrüm Aydin, Lisa M. Olsson, Wilhelm Sjöland, Marcus Henricsson, Annika Lundqvist, Hanns-Ulrich Marschall, Rutger Franken, Arnold van de Laar, Victor Gerdes, Abraham S. Meijnikman, Dag Hofsø, Albert K. Groen, Jøran Hjelmesæth, Max Nieuwdorp, Fredrik Bäckhed

**Affiliations:** a Wallenberg Laboratory and Department of Molecular and Clinical Medicine, Institute of Medicine, Sahlgrenska Academy, University of Gothenburg, Gothenburg S-413 45, Sweden; b Department of Internal and Vascular Medicine, Amsterdam UMC, Amsterdam, the Netherlands; c Department of Internal Medicine, Spaarne Gasthuis, Hoofddorp, the Netherlands; d Department of Surgery, Spaarne Hospital, Hoofddorp, the Netherlands; e Department of Endocrinology, Obesity and Nutrition, Vestfold Hospital Trust, Tønsberg, Norway; f Experimental Vascular Medicine, Amsterdam UMC, Amsterdam, the Netherlands; g Department of Endocrinology, Morbid Obesity and Preventive Medicine, Institute of Clinical Medicine, University of Oslo, Norway; h Novo Nordisk Foundation Center for Basic Metabolic Research, Faculty of Health Sciences, University of Copenhagen, Copenhagen DK-2200, Denmark; i Region Västra Götaland, Sahlgrenska University Hospital, Department of Clinical Physiology, Gothenburg, Sweden

**Keywords:** 6α-hydroxylated bile acids, Diabetes remission, Postprandial response, Roux-en-Y gastric bypass

## Abstract

**Background:**

Bariatric surgery is an effective treatment option for obesity and provides long-term weight loss and positive effects on metabolism, but the underlying mechanisms are poorly understood. Alterations in bile acid metabolism have been suggested as a potential contributing factor, but comprehensive studies in humans are lacking.

**Methods:**

In this study, we analysed the postprandial responses of bile acids, C4 and FGF19 in plasma, and excretion of bile acids in faeces, before and after bariatric surgery in patients (n = 38; 74% females) with obesity with or without type 2 diabetes from the BARIA cohort.

**Findings:**

We observed that total fasting plasma bile acid levels increased, and faecal excretion of bile acids decreased after surgery suggesting increased reabsorption of bile acids. Consistent with increased bile acid levels after surgery we observed increased postprandial levels of FGF19 and suppression of the bile acid synthesis marker C4, suggesting increased FXR activation in the gut. We also noted that a subset of bile acids had altered postprandial responses before and after surgery. Finally, fasting plasma levels of 6α-hydroxylated bile acids, which are TGR5 agonists and associated with improved glucose metabolism, were increased after surgery and one of them, HDCA, covaried with diabetes remission in an independent cohort.

**Interpretation:**

Our findings provide new insights regarding bile acid kinetics and suggest that bariatric surgery in humans alters bile acid profiles leading to activation of FXR and TGR5, which may contribute to weight loss, improvements in glucose metabolism, and diabetes remission.

**Funding:**

10.13039/501100009708Novo Nordisk Fonden, 10.13039/501100001674Leducq Foundation, 10.13039/501100003793Swedish Heart-Lung Foundation, 10.13039/501100004063Knut and Alice Wallenberg Foundation, the ALF-agreement, 10.13039/501100001826ZonMw.

**Research in context**

**Evidence before this study**

Obesity is becoming a major health issue worldwide and can cause diseases like heart disease, type 2 diabetes, and non-alcoholic fatty liver disease. Bariatric surgery is currently the most effective long-term treatment for obesity and its related health problems, although the exact reasons for its success are not fully understood. Initially, it was believed that weight loss from the surgery was mainly due to reduced nutrient absorption, as the procedure makes the stomach smaller or bypasses parts of the intestine, which also helps improve blood sugar levels. However, changes in bile acid composition have been hypothesized to mediate the metabolic improvements seen after bariatric surgery. Bile acid levels depend on their production in the liver, bioconversion by gut bacteria, reabsorption from the intestines, and excretion in faeces. Changes in this balance can affect how bile acids signal through specific receptors, which can impact blood glucose and lipid metabolism. Human studies have shown that bile acid levels in the blood change after surgery, both when fasting and after a meal. However, these studies often have limitations, such as few participants, differences in glucose levels before surgery, and lack of long-term follow-up.

**Added value of this study**

Here we showed that total fasting plasma bile acid levels increased dramatically after surgery, while faecal excretion of bile acids decreased after surgery, thus suggesting increased reabsorption of bile acids. Consistent with increased bile acid levels after surgery we observed increased postprandial levels of FGF19 and suppression of the bile acid synthesis marker C4, suggesting increased FXR activation in the gut. We also noted that a subset of bile acids had altered postprandial responses before and after surgery. Interestingly, fasting plasma levels of 6α-hydroxylated bile acids, which are TGR5 agonists and associated with improved glucose metabolism, were increased after surgery and one of them, HDCA, covaried with diabetes remission in an independent cohort.

**Implications of all the available evidence**

Thus, we here provide clear evidence for that bariatric surgery modulates bile acid composition and kinetics, consistent with increased FXR activation, which has been implicated to mediate metabolic improvements following surgery. Furthermore, we demonstrate that HDCA may contribute to T2D remission, which is consistent with pre-clinical data.

## Introduction

Obesity is a growing global health problem associated with metabolic diseases such as cardiovascular disease, type 2 diabetes (T2D), and nonalcoholic fatty liver disease.^1^^,^^2^ Bariatric surgery, including Roux-en-Y gastric bypass (RYGB) and vertical sleeve gastrectomy, is currently the most effective treatment option that provides a long-term effect on obesity and related co-morbidities but the mechanisms behind the positive effects on weight loss and metabolic parameters are still not completely understood.^3^^,^^4^ Initially, it was thought that the main effect on weight loss was caused by malabsorption of nutrients due to the restricted size of the stomach and/or the bypass of parts of the intestine, with subsequent improvement in glucose homeostasis. The role of weight loss on metabolic improvement after bariatric surgery is ambiguous and some studies have shown that positive effects on glucose homeostasis can occur rapidly before significant weight loss,^5^, ^6^, ^7^, ^8^, ^9^ or that the initial calorie restriction after surgery is responsible for the improvements in insulin sensitivity,^10^ while others indicate that the clinically important effects after surgery are dependent on weight loss.^11^^,^^12^

Alterations in bile acid homeostasis have been suggested as a contributing factor for the metabolic improvement after surgery, along with alterations in the gut microbiota, but a clear consensus has not yet been reached.^13^, ^14^, ^15^, ^16^, ^17^ Homeostasis of bile acids is dependent on their synthesis in the liver, microbial metabolism in the gut, reuptake from the intestine and excretion via faeces.^18^, ^19^, ^20^ Changes in bile acid homeostasis can influence their signalling via bile acid receptors, such as farnesoid X receptor (FXR) and Takeda G-protein coupled receptor 5 (TGR5), which can modulate changes in glucose and lipid metabolism.^21^ Mouse models have shown that both FXR and TGR5 are important for the positive effects on glucose homeostasis after bariatric surgery,^22^, ^23^, ^24^, ^25^, ^26^, ^27^ which supports the assumption that changes in bile acid signalling contribute to the metabolic improvements after bariatric surgery although the mechanisms are not fully understood.

Several human studies have shown changes in plasma or serum bile acid levels in the fasting state after RYGB as reviewed by Cole et al.,^13^ and some studies have also shown alterations in the postprandial state.^28^^,^^29^ However, common limitations in human studies are low numbers of study participants, large interindividual variations in glucose status before surgery and lack of longitudinal follow-ups.^13^ Hence, there is an urge for additional human studies on bile acid kinetics before and after bariatric surgery. In this study, we therefore investigated postprandial bile acid kinetics after a mixed-meal test in the same patients before and one year after bariatric surgery.

## Methods

### Study cohorts

In our study population, we included the first 38 patients (22 without T2D and 16 with T2D) from the BARIA cohort, which has previously been described,^30^ who had completed mixed-meal tests before bariatric surgery and at the one-year follow up visit. Clinical parameters measured before and after surgery are presented in Table 1 and sex was self-reported. All 22 patients without T2D underwent laparoscopic Roux-en-Y gastric bypass (RYGB), and in the group with T2D, 10 patients underwent RYGB, and 6 mini gastric bypass (MGB). The surgical procedures are similar and bypass a large part of the stomach. The RYGB is a double-anastomosis surgery with 150 cm of the small intestine bypassed and the MGB is a single-anastomosis bypass, with 200 cm of the small intestine bypassed.^31^ Inclusion criteria were men and women with obesity between 18 and 65 years of age with a body mass index (BMI) ≥40 kg/m^2^, or BMI ≥ 35 kg m/2 with obesity-related comorbidity. Exclusion criteria were primary lipid disorder, known genetic basis for insulin resistance or glucose intolerance, psychiatric conditions, coagulation disorders, uncontrolled hypertension (blood pressure > 150/95 mmHg), renal insufficiency (creatinine > 150 μmol/L), excessive alcohol intake (>14 units/week), pregnancy, and breastfeeding. The patients were not treated with ursodeoxycholic acid after surgery. The patients in this cohort were recruited consecutively from the obesity clinic and can be considered representative for a population with obesity.

**Table 1:** Clinical parameters before and after surgery.

Clinical parameters	PRE surgery	POST surgery	p
Age [years]	50 (40.5–54)		
Female/Male	28/10		
Surgery type [RYGB/MGB]	32/6		
T2D [Yes/No]	16/22	4/34	0.001
Cholecystectomi [Yes/No]	4/34	5/33	1
Weight [kg]	114.2 (106.8–126.6)	80.5 (73.6–93.9)	8e-08
BMI [kg/m^2^]	39 (36–40.9)	27.9 (25.7–30)	7.3e-12
Weight loss [%]		25.8 (23.3–33.1)	
ALP [U/L]	78 (59.8–91.5)	85 (71.5–97.5)	0.014
ALT [U/L]	23.5 (19–28.8)	26.5 (19.8–34.2)	0.64
AST [U/L]	22 (19.2–26)	23 (19.8–28.2)	0.36
Bilirubin [U/L]	8 (6–11)	9 (7–12)	0.039
GGT [U/L]	24.5 (22–40.5)	17.5 (14.2–23.8)	0.00021
SBP [mmHg]	131 (122–137)	119.5 (112.2–127.8)	0.0021
DBP [mmHg]	80 (78–86)	77 (72.2–81.8)	0.0065
Chol [mmol/L]	4.8 (3.9–5.9)	4.4 (3.8–5.2)	0.025
LDL [mmol/L]	3 (2.4–4)	2.6 (2.1–3.1)	0.0014
HDL [mmol/L]	1.1 (1–1.3)	1.4 (1.3–1.6)	0.00083
CRP [mg/L]	5.8 (2.2–9.7)	1 (0.6–1.6)	2.3e-06
Triglycerides [mmol/L]	1.5 (1.2–1.9)	1 (0.7–1.6)	4.9e-05
Hb [g/dL]	8.8 (8.3–9.2)	8.6 (8–9.1)	0.0091
Fasting glucose [mmol/L]	6 (5.4–7.4)	5.2 (4.9–6)	2.6e-06
Fasting insulin [pmol/L]	82 (50.3–131)	30 (18.2–45.2)	1.3e-07
Fasting C-peptide [unit]	0.8 (0.6–1)	0.4 (0.3–0.6)	4.5e-07
HbA1c [%]	5.8 (5.4–7)	5.4 (5.1–5.6)	2.6e-06
HOMA-IR	2 (1.1–3.1)	0.7 (0.4–1)	9.3e-10
T2D duration [years]	4.5 (1–7.8)		
Number of medications [N]	2.5 (1–5.8)	1 (1–3)	0.00015
T2D medication [Yes/No]	14/24	4/34	0.004
Metformin [Yes/No]	13/25	4/34	0.008
Gliclazide [Yes/No]	3/35	0/38	0.2
Insulin [Yes/No]	2/36	0/38	0.5
GLP1 agonist [Yes/No]	2/36	0/38	0.5
Statins [Yes/No]	9/29	4/34	0.1
PPI [Yes/No]	5/33	11/27	0.2
SSRI [Yes/No]	5/33	4/34	1

Median (Q1–Q3), p-values indicate differences between PRE and POST surgery using Wilcoxon signed-rank test for continuous variables, and NcNemar test for categorical variables.

The study protocol was approved by the Ethical Review Board of the Academic Medical Center, Amsterdam (approval code: NL55755.018.15), and all included patients have provided informed consent.

The results were validated in 39 patients with obesity and T2D undergoing RYGB from another randomised controlled bariatric surgery cohort (Oseberg), in which fasting plasma bile acids were measured. Inclusion criteria for this cohort were: age over 18 years, a BMI over 33 kg/m^2^ or a previously verified BMI over 35 kg/m^2^ and with T2D defined as Hb1Ac ≥ 6.5% (48 mmol/mol) or > ≥6.5% (48 mmol/mol) using antidiabetic medications. Diabetes remission in this cohort was defined by Hb1Ac ≤ 6.0% (42 mmol/mol) without glucose-lowering medications. The validation cohort only includes patients with T2D and is less representative for a population with obesity. The study protocol was approved by the Regional Committees for Medical and Health Research Ethics in Norway (2012/1427/REK sør-øst B) and all included patients have provided informed consent.^32^

### Mixed-meal test

A mixed-meal test was performed in the BARIA cohort before surgery (spanning from 2 weeks to 3 months before surgery) and one year after surgery as described previously.^30^ The patients fasted for a minimum of 9 h and then received a meal consisting of two 125 ml Nutridrink compact (Nutricia®), containing 23.3 g fat, 74.3 g carbohydrates (38.5 g sugar) and 24.0 g protein in total. The baseline (0 min) was the moment when the patient had consumed the entire meal. Blood was drawn at baseline, 10, 20, 30, 60, 90, and 120 min and glucose, insulin, fibroblast growth factor 19 (FGF19), bile acids and 7α-hydroxy-cholesten-4-one (C4) were analysed.

### Analyses of glucose and insulin

Glucose and insulin measurements were performed on plasma collected in lithium heparin tubes. Glucose was determined by the ISO 14155:2020, IDT certified diagnostic laboratory in the University Medical Center Amsterdam. Insulin was measured using the Immunometric assay, Luminescence, Atellica IM, (Siemens).

### Analyses of FGF19

FGF19 was analysed in plasma using Human FGF-19 Quantikine ELISA kit (DF 1900, R&D Systems, Minneapolis, MN, USA), according to the manufacturer’s instructions. Plasma FGF19 concentrations are presented in pg/mL.

### Faecal collection

Faeces were collected for 24 h one day before the mixed-meal test (before surgery and one year after surgery). The 24-h faecal sample was weighed and mixed with an equal amount of water and stored at −80 °C. Aliquots of 40–110 mg were used for bile acid analysis. Faecal bile acid concentrations were measured in all 38 individuals, but total 24-h faecal weights were missing for some individuals. Therefore, paired statistical analysis of bile acid levels in the whole 24-h sample before and after surgery could only be done in the 30 individuals with 24-h faecal weights for both timepoints.

### Bile acid and C4 analysis

Bile acid analyses were performed on faeces, and bile acid and C4 analyses were performed on plasma before and after bariatric surgery. Faecal samples were extracted with methanol, containing d4-TCA, d4-GCA, d4-GCDCA, d4-GLCA, d4-UDCA, d4-CA, d4-CDCA, d4-LCA (2.5 μM of each). The samples were homogenized with ceramic beads for 2 × 10 min using the Qiagen Tissuelyser II. After 10 min of centrifugation at 20,000*g*, samples were diluted 50x and 250x in methanol:water 1:1. Samples were run twice, once at 50x dilution, and once at 250x dilution. All bile acids apart from DCA and sulfated bile acids were evaluated at 50x dilution, while DCA and sulfated bile acids were evaluated at 250x dilution to avoid effects from detector saturation.

Plasma samples (50 μL) were extracted with 10 volumes of methanol with the same deuterium-labelled standards as above (50 nM of each). After 5 min of vortexing at 1500 rpm and 15 min of centrifugation at 3000*g*, supernatants were evaporated using a stream of nitrogen and reconstituted in 200 μL methanol:water [1:1]. 5 μL of the samples were used for the bile acid analysis. Bile acids were analysed using ultra-performance liquid chromatography-tandem mass spectrometry (UPLC-MS/MS) according to previous work.^28^ Briefly, after injection the bile acids were separated on a C18 column (1.7 μm, 2.1 × 100 mm; Kinetex, Phenomenex, USA) using water with 7.5 mM ammonium acetate and 0.019% formic acid (mobile phase A) and acetonitrile with 0.1% formic acid (mobile phase B). The chromatographic separation started with 1-min isocratic separation at 20% B. The B-phase was then increased to 35% during 4 min. During the next 10 min the B-phase was increased to 100% and held at 100% for 3.5 min before returning to 20%. The total runtime was 20 min. Bile acids and C4 were detected using multiple reaction monitoring (MRM) in negative mode (C4 in positive mode) on a QTRAP 5500 mass spectrometer (Sciex, Concord, Canada) and quantification was made against appropriate deuterated internal standards with adjustments by using external individual standard curves.

### Calculations and statistical analysis of bile acid kinetics from the mixed-meal test

Maximum concentration (MC) for each bile acid was determined as the highest value during the mixed-meal test. Maximum rise (MR) for each bile acid was calculated by subtracting the fasting concentration (FC) from the MC during the mixed-meal test to estimate the magnitude of the increase. The percentage of the maximum rise (MR/FC ∗100) was calculated to estimate the magnitude of the response in relation to the fasting value for each bile acid. Thus, the responses of different bile acids could be compared although the absolute levels of specific bile acids vary and there is a large interindividual variation. To determine if a bile acid showed a response or not, we dichotomised the bile acids into categories; bile acids with a median MR/FC ∗100 > 100 (indicating a doubled fasting concentration in more than half of the patients) were categorized as “responsive”, and bile acids with a median MR/FC ∗100 < 100 as “non-responsive”.

To investigate if there was a faster response in plasma bile acid levels following bariatric surgery, we determined the timepoint for the maximum concentration (TMC). If the TMC occurred at 30 min or earlier in more than 70% of the patients, the bile acid was categorized as having an “early response”; if TMC occurred later, it was categorized as having a “late response”. We also evaluated a different cut-off for the early response based on that TMC should occur at 15 min in more than 70% of the patients. When applying the 15 min cut-off, there was only one bile acid that showed an early response, and our subsequent interpretation of the data is based on the 30 min cut-off.

Data distribution was assessed with Shapiro–Wilk test and showed that concentrations of plasma and faecal bile acids and the majority of the clinical parameters were not normally distributed; hence, non-parametric tests were used. Changes in concentrations were tested using Wilcoxon signed-rank test. Change over surgery linked with covariation of T2D remission in the Oseberg trial were evaluated using LongDat v.1.1.^33^ Clinical parameters were tested using Wilcoxon signed-rank test for continuous variables and with NcNemar test for categorical variables. Correlation between C4 and total faecal bile acid concentration was determined with two-tailed Spearman’s rank correlation test. Differences in glucose and insulin levels between the nonT2D group and the T2D group before or after surgery were tested using Multiple Mann–Whitney tests.

### Role of funders

None of the funders had any role in study design, data collection, data analyses, interpretation, or writing the report.

## Results

### Study cohort

Clinical parameters measured in patients from the BARIA cohort before and one year after surgery are presented in Table 1. All 38 patients displayed significant weight loss one year after surgery. The median weight loss was 25.8% and BMI decreased from 39.0 kg/m^2^ to 27.9 kg/m^2^ (p < 0.0001) (Table 1). There were also beneficial changes in other parameters such as blood pressure and C-reactive protein (CRP) levels in plasma, and improved lipid profiles with decreased plasma levels of cholesterol, low-density lipoprotein (LDL) and triglycerides (TG), and increased levels of high-density lipoprotein (HDL) after surgery. Liver function tests such as alkaline phosphatase (ALP), alkaline transaminase (ALT), aspartate aminotransferase (AST), bilirubin and gamma-glutamyltransferase (GGT) were all within the normal range both before and after surgery.

### Total fasting bile acid levels in plasma are increased, and faecal bile acid excretion is decreased after bariatric surgery

**Fig. 1:**
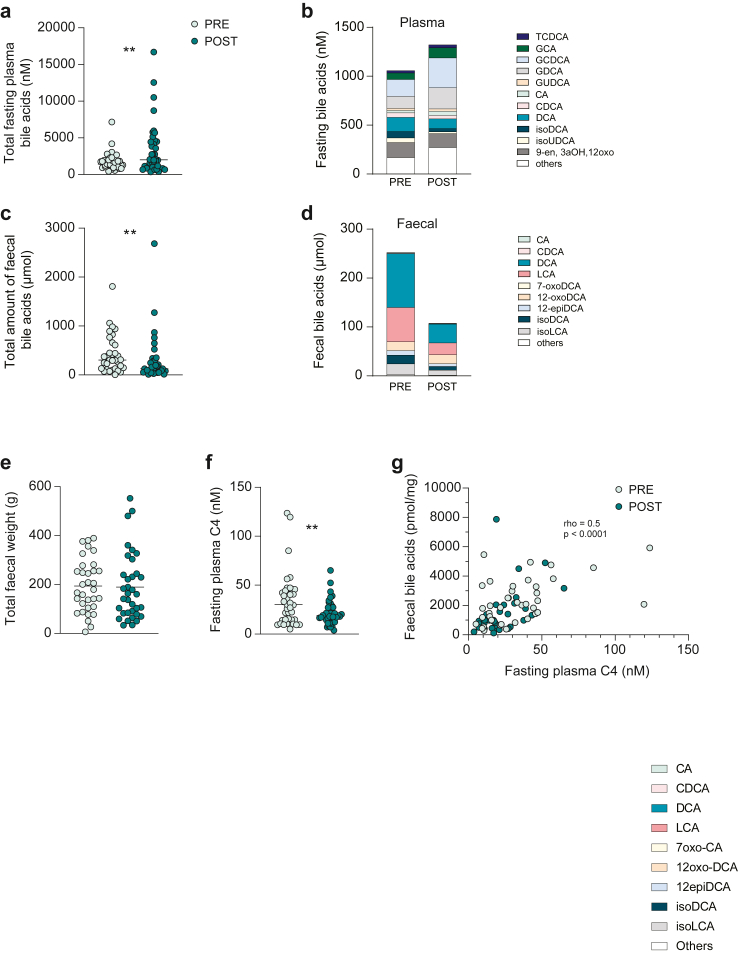
**Alterations in fasting plasma bile acids and faecal bile acid excretion after bariatric surgery.** (a) Total fasting plasma bile acid concentration presented in nM. (b) Fasting plasma bile acid composition. (c) Total amount of faecal bile acids from 24 h sampling presented in μmol. (d) Faecal bile acid composition. (e) Total faecal weight from 24 h sampling presented in g. (f) Fasting plasma C4 concentration. (g) Spearman’s rank correlation analysis between faecal bile acid concentration in pmol/mg and fasting plasma C4 levels. Before surgery, PRE, (light green circles) and after surgery, POST (dark green circles). ∗p < 0.05, ∗∗p < 0.01, indicate differences before and after surgery using Wilcoxon signed-rank test, n = 38 samples per group in panel a, b, d, f, g and n = 33 samples for PRE and n = 35 samples for POST in panel c and e. The lines indicate median in panel a, c, e, f. CA, cholic acid; CDCA, chenodeoxycholic acid; DCA, deoxycholic acid; GCA, glyco-cholic acid; GCDCA, glyco-chenodeoxycholic acid; GDCA, glyco-deoxycholic acid; GUDCA, glyco-ursodeoxycholic acid; LCA, lithocholic acid; isoDCA, iso-deoxycholic acid (3β-deoxycholic acid); isoLCA, iso-lithocholic acid (3β-lithocholic acid); isoUDCA, iso-ursodeoxycholic acid (3β-ursodeoxycholic acid); TCDCA, tauro-chenodeoxycholic acid; 7-oxoCA, 7-oxocholic acid; 12-epiDCA, 12-epideoxycholic acid (12β-deoxycholic acid); 12-oxoDCA, 12-oxodeoxycholic acid. “Others” represent the sum of bile acids contributing to less than 2% of the total pool for plasma, and less than 1% of the total pool for faeces.

First, we analysed bile acids in fasting plasma samples to determine how surgery affected fasting levels of bile acids and observed increased levels of total plasma bile acids after surgery (Fig. 1a, Supplementary Table S1). Fasting concentrations of bile acids generally reflect the steady state associated with the repeated enterohepatic cycling of bile acids. This increase was mainly driven by primary bile acids and among individual bile acids glyco-chenodeoxycholic acid (GCDCA), glyco-cholic acid (GCA) and CA were significantly increased (Fig. 1b and Supplementary Table S1). There were also increased levels of the 6-hydroxylated bile acids hyodeoxycholic acid (HDCA), hyocholic acid (HCA), and the “murine” bile acids: tauro-alpha muricholic acid (TαMCA) and tauro-omegamuricholic acid (TωMCA), while the iso-bile acids: iso-ursodeoxycholic acid (isoUDCA), iso-deoxycholic acid (isoDCA) and iso-lithocholic acid (isoLCA), decreased after surgery (Supplementary Table S1).

Next, we evaluated excretion of bile acids by analysing faecal samples collected for 24 h and found that the total amount of faecal bile acids decreased after surgery (Fig. 1c). The most abundant faecal bile acids were, as expected, DCA and LCA, which are produced by microbial 7α-dehydroxylation of CA and CDCA, respectively (Fig. 1d). However, levels of LCA and iso-LCA decreased after surgery, while DCA levels were unchanged. Some low-abundant bile acids (TCDCA and GLCA) were also decreased after surgery (Supplementary Table S2). Note that 24-h faecal weights were similar before and after surgery, thus, faecal output did not change after surgery (Fig. 1e, Supplementary Table S2).

Loss of bile acids via faeces is compensated by synthesis of new bile acids from cholesterol in the liver,^34^ which is strongly correlated with plasma C4, a stable intermediate in bile acid synthesis and an established marker for bile acid synthesis.^35^ In agreement with increased plasma and decreased faecal bile acids, suggesting increased absorption and reduced excretion of bile acids from the gut after surgery, we observed decreased levels of fasting C4 in plasma after surgery (Fig. 1f). Accordingly, fasting C4 was strongly correlated with total faecal bile acid concentration (r = 0.5; p < 0.0001; Fig. 1g).

### Postprandial bile acid kinetics is altered after bariatric surgery

Next, we analysed the bile acid levels, C4 and FGF19 in plasma after mixed-meal tests, before and after surgery, to assess how the rearrangement of the gut affects the postprandial responses. In contrast to fasting levels, postprandial bile acid levels mainly reflect the absorption of the intestinal contents after the meal.

**Fig. 2:**
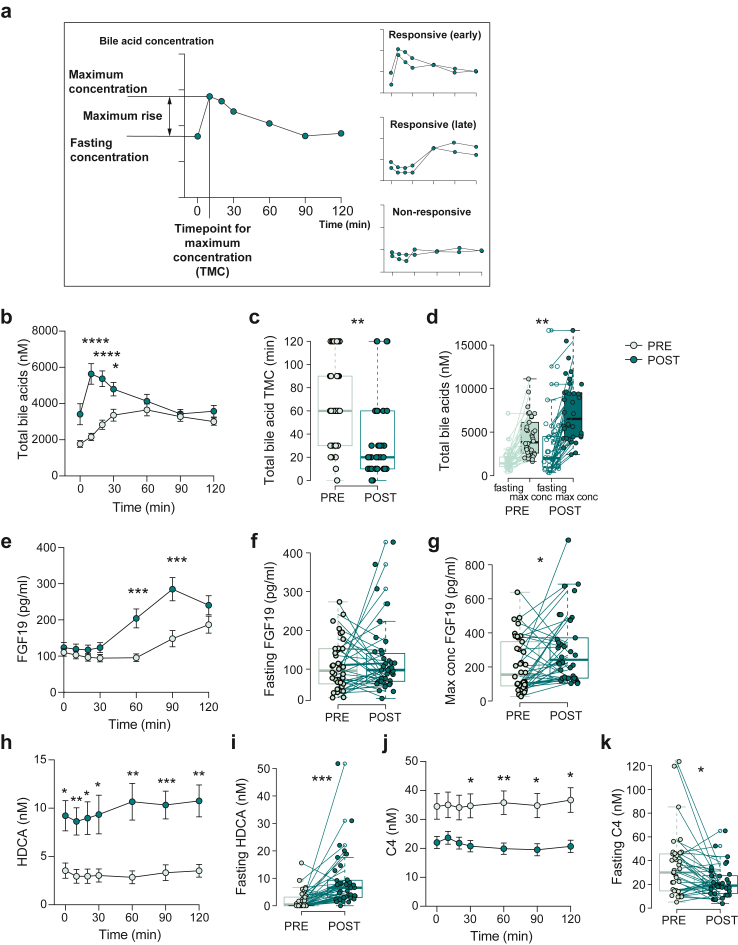
**The impact of bariatric surgery on bile acid kinetics.** (a) Schematic figure of definitions describing kinetics of plasma bile acid levels during the mixed-meal test. The different kinetic patterns are classified as “responsive” (early or late) or “non-responsive”. (b) Total bile acid levels, as an example of “early-response” kinetics, presented as mean and standard error of mean (SEM) in nM at the different timepoints during the mixed-meal test before (PRE) and after (POST) surgery. (c) Timepoint of maximum concentration (TMC) for total bile acids presented in min before and after surgery. (d) Fasting and max concentration of total bile acids before and after surgery. (e) Plasma FGF19 levels, as an example of “late-response” kinetics, presented as mean ± SEM at the different timepoints during MMT before and after surgery. (f) Fasting levels and (g) maximum concentrations of FGF19 in pg/ml during MMT before and after surgery. (h) Levels of HDCA as an example of “non-responsive” kinetics, presented as mean ± SEM at the different timepoints during mixed-meal tests before and after surgery. (i) Fasting levels of HDCA before and after surgery presented. (j) The bile acid synthesis marker C4 as another example of “non-responsive” kinetics presented as mean ± SEM at the different timepoints during mixed-meal tests before and after surgery. (k) Fasting levels of C4 before and after surgery. ∗Padj < 0.05, ∗∗Padj < 0.01, ∗∗∗Padj < 0.001 indicate differences between PRE and POST surgery using Wilcoxonsigned-rank test, n = 38 samples per group. Mean ± SEM are shown in the graphs in panel b, e, h, j. Boxes show median and interquartile ranges (IQR); whiskers specify ±1.5∗IQR from box’s quartile in panel c, d, f, g, i, and k. Before surgery (PRE; light green) and after surgery (POST; dark green). Samples from the same patient are connected with lines in panel d, f, g, i and k.

To disentangle changes in the kinetics, we first determined the maximum concentration during the mixed-meal test and the maximum rise in concentration for each bile acid as well as for C4 and FGF19 for each individual (Fig. 2a). To study individual responses of each bile acid, we divided the maximum rise by the fasting concentration for the specific bile acid in each individual and categorized the bile acids as “responsive” or “non-responsive” based on their increase in relation to the fasting concentration (see Method). Total bile acid levels, and all the taurine- and glycine-conjugated bile acids, except GHDCA, showed a postprandial response before surgery, while unconjugated bile acids, C4 and FGF19 did not (Supplementary Table S1). In contrast, after surgery, some unconjugated bile acids that were categorized as “non-responsive” before surgery showed clear postprandial responses (Supplementary Table S1). Furthermore, we could define specific changes in the postprandial response: some bile acids reached maximum concentrations earlier and then declined during the mixed-meal test, while others reached maximum concentration late during the test (Fig. 2a). These “early response” bile acids reached the timepoint for maximum concentration (TMC) at 30 min or earlier in more than 70% of the patients. Using this definition, total bile acid levels showed an “early response” after surgery, and the levels were significantly higher at 10, 20 and 30 min compared with before surgery (Fig. 2b). Specific bile acids with an “early response” after surgery were taurine-conjugated primary (TCA and TCDCA) and secondary bile acids (TDCA, TLCA and TUDCA) as well as glycine-conjugated bile acids (GCA and GDCA), consistent with that conjugated bile acids are efficiently absorbed from the small intestine after being secreted from the gallbladder after the meal challenge (Supplementary Table S1, and Fig. 2c for total bile acids). The median maximum concentration of total bile acids increased from 3829 nM to 6517 nM (Supplementary Table S1 and Fig. 2d), indicating that in addition to an earlier postprandial response there is also increased absorption of bile acids after surgery. FGF19, unconjugated bile acids (CA, CDCA, UDCA, DCA) and glycine-conjugated bile acids (GCDCA, GUDCA, GLCA) showed a “late response” after surgery with a maximum concentration at the end of the mixed-meal test and a median TMC of 90 min (Fig. 2e and Supplementary Table S1).

Importantly, fasting concentrations of several bile acids (such as GDCA, TDCA, TCA, CDCA and DCA) as well as FGF19, were not altered after surgery, but by evaluating their maximum concentrations, we were able to capture differences that we would not have observed if only fasting concentrations had been analysed (Fig. 2f and G and Supplementary Table S1).

However, for some of the “non-responsive” bile acids, such as the 6α-hydroxylated bile acids, HDCA, GHDCA, HCA and GHCA, there were increases in the fasted state after surgery, and their levels were high throughout the test, which indicate that their levels might be chronically elevated and that the effect of surgery could be captured without a mixed-meal challenge (Fig. 2h and I and Supplementary Table S1). Similarly, there were no postprandial responses in C4 levels neither before nor after surgery, but the C4 level decreased after surgery (Fig. 2j and k and Supplementary Table S1).

### The effect of bariatric surgery on metabolic parameters in patients with or without T2D at surgery

We next investigated if surgical effects on bile acid levels and kinetics differed in patients with or without T2D. Patients with T2D at surgery lost less weight (23.7% in T2D versus 30.7% in nonT2D; p < 0.001, [two-tailed Student’s t-test]) and had higher BMI (28.7 kg/m^2^ in T2D versus 26.7 kg/m^2^ in nonT2D, p = 0.03, [two-tailed Student’s t-test]) after surgery compared to patients without T2D, although BMI was similar between the groups at surgery (Supplementary Table S3).

Fasting blood glucose and insulin also decreased in both groups after surgery, although the levels were still higher in the group with T2D compared to the group without T2D. Consistent with improved glucose metabolism, fasting HbA1c and C-peptide also decreased after surgery (Supplementary Table S3). As expected, glucose levels during the MMTs were substantially higher in the patients with T2D both before and after surgery, but both groups showed an earlier peak and more rapid reduction after 30 min (Supplementary Fig. S1a). Insulin responses before surgery were rather similar in the T2D and nonT2D groups, and after surgery, the peak of insulin occurred earlier in both groups and decreased after 60 min, suggesting an incretin effect (Supplementary Fig. S1b). Interestingly, 12 of the 16 patients with T2D at surgery did not require diabetes medication after 12 months, emphasizing the strong effect of bariatric surgery on T2D remission, which is in line with previous findings.^32^^,^^36^^,^^37^

### The effect of bariatric surgery on bile acid kinetics is blunted in patients with T2D at surgery

**Fig. 3:**
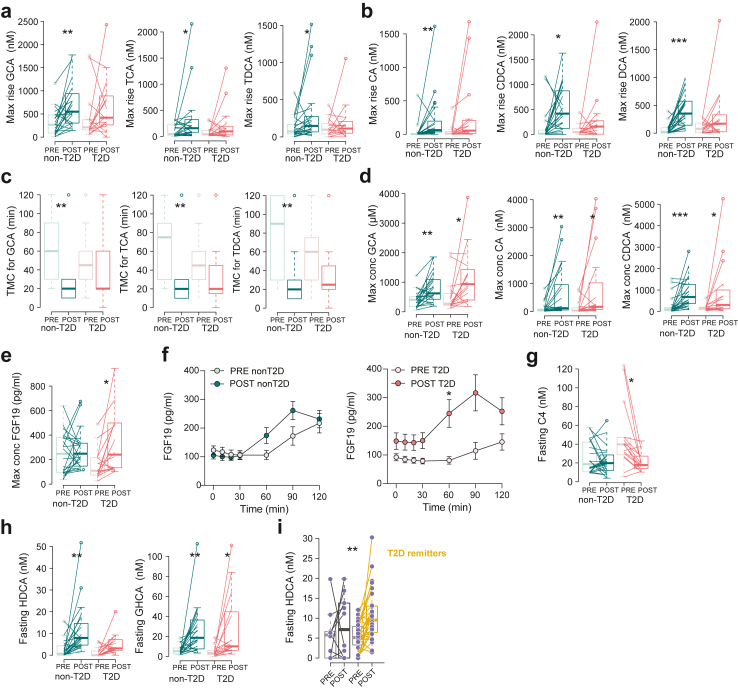
**Type 2 diabetes specific alterations in plasma bile acid kinetics after surgery.** (a and b) Maximum rise of “early response” bile acids GCA, TCA and TDCA (a) and “late response” bile acids CA, CDCA and DCA (b), before (PRE) and after (POST) surgery in patients without T2D (nonT2D) or with pre-surgery T2D (T2D). nonT2D group; green colors, T2D group; pink colors, PRE; light color, POST; dark color. (c) Timepoint of maximum concentration (TMC) during the mixed-meal tests of “early response” bile acids GCA, TCA and TDCA, before and after surgery in patients without or with pre-surgery T2D. (d) Maximum concentration of GCA, CA and CDCA during the mixed-meal tests, before and after surgery in patients without or with pre-surgery T2D. (e) Maximum concentration of plasma FGF19 during the mixed-meal tests, before and after surgery in patients without or with pre-surgery T2D. (f) Plasma FGF19 levels, presented as mean ± SEM at the different timepoints during the mixed-meal tests in patients without T2D (first panel) or with pre-surgery T2D (second panel), before and after surgery. (g) Fasting concentration of plasma C4, before and after surgery in patients without or with pre-surgery T2D. (h) Fasting concentration of 6-alpha hydroxylated bile acids HDCA and GHCA, before and after surgery in patients without or with pre-surgery T2D. (i) Fasting concentration of HDCA in another bariatric surgery cohort with only pre-surgery T2D patients before and after surgery divided according to T2D remission after 1 year (no remission, black lines; T2D remission, yellow lines). ∗Padj < 0.05, ∗∗Padj < 0.01, ∗∗∗Padj < 0.001 indicate differences between PRE and POST surgery using Wilcoxon signed-rank test, n = 22 for the nonT2D group and n = 16 for the T2D group. Boxes show median and interquartile ranges (IQR); whiskers specify ±1.5∗IQR from box’s quartile in panel a–e, g, h and i. Mean ± SEM are shown in the graphs in panel f. Samples from the same patient are connected with lines in a and b, d and e, and g–i.

Alterations in bile acid kinetics after surgery differed in patients with and without T2D at surgery. The T2D group had a blunted effect on the postprandial response of bile acids after surgery and the increase in maximum rise that we observed after surgery for both “early response bile acids” (such as GCA, TCA and TDCA) (Fig. 3a and Supplementary Table S4), and “late response” bile acids (such as CA, CDCA and DCA) (Fig. 3b and Supplementary Table S4), were only significant in the nonT2D group. Similarly, TMC for the “early response” bile acids only decreased after surgery in patients without T2D at surgery (Fig. 3c). However, maximum concentrations of GCA, which was classified as an “early response” bile acid, and CA and CDCA, which were classified as “late response” bile acids, increased in both groups after surgery (Fig. 3d and Supplementary Table S4), indicating that bariatric surgery may lead to alterations in bile acid kinetics also in the T2D group but the heterogeneity of this group makes it difficult to capture significant changes in the postprandial response i.e., maximum rise or TMC. Finally, since 6 of the patients in the T2D group underwent MGB and not RYGB, we investigated if this could be a source of heterogeneity in responses in this group, but the results were consistent even if the 6 patients were excluded from the group (Supplementary Table S5).

### Postprandial response in FGF19 and fasting C4 levels are normalized in patients with T2D at surgery

Contrary to the blunted effect on bile acid kinetics in the T2D group, the changes in FGF19 and C4 after surgery were mainly driven by changes in the T2D group. Fasting FGF19 levels were not altered in any of the groups after surgery, however, the maximum concentration of FG19 increased in the T2D group but not in the nonT2D group (Fig. 3e and Supplementary Table S4). When comparing the postprandial FGF19 response curves, we observed a dampened FGF19 response in the T2D group before surgery, and after surgery, the FGF19 response was normalized especially in patients with T2D at surgery (Fig. 3f).

Similarly, the decrease in fasting C4 levels after surgery was only significant in the group with T2D at surgery and this was due to normalization of the higher levels before surgery, while there was no difference in the nonT2D group (Fig. 3g and Supplementary Table S4).

### Increased fasting levels of HDCA is linked to T2D remission

6α-hydroxylated bile acids, HDCA and GHCA, were increased after surgery in the BARIA cohort (Fig. 3h). Since HDCA was “non-responsive” and did not change after a meal test, we sought to validate our findings in an independent cohort. To this end, we included 39 patients with T2D from a second bariatric surgery cohort,^32^^,^^36^ where fasting bile acids were analysed, and we observed that HDCA levels were increased after surgery here as well (Fig. 3i). Furthermore, covariance analysis showed that HDCA covaried with diabetes remission in the validation cohort (Supplementary Table S6) and the largest effect of surgery on HDCA levels was found in patients with diabetes remission (Fig. 3i), suggesting that HDCA levels potentially can predict T2D remission and be involved in the metabolic improvements after RYGB.

## Discussion

Here we analysed the levels and composition of faecal and plasma bile acids, as well as plasma bile acid kinetics during mixed-meal tests, in patients with obesity with or without T2D before and 12 months after bariatric surgery. We showed that individual bile acids have different postprandial responses, and that bariatric surgery modulates the kinetics of specific bile acids. Furthermore, we showed that the effect of bariatric surgery on metabolic parameters and bile acid kinetics is partially blunted in patients with T2D and that HDCA is associated with remission of T2D. Finally, our data suggests that alterations in bile acid profiles are consistent with activation of bile acid receptors that have previously been identified in mouse models.^38^

Taurine- or glycine-conjugated bile acids demonstrated clear postprandial responses before surgery, which is consistent with the common concept of conjugated bile acids being absorbed via the ileal bile acid transporter (IBAT) in the distal ileum before entering the enterohepatic circulation and transported to the liver via the portal vein.^18^^,^^39^ Taurine-conjugated bile acids are more hydrophilic than glycine-conjugated bile acids and rely almost exclusively on active absorption via IBAT, while glycine-conjugated bile acids can also be absorbed via passive diffusion,^18^ and this might lead to differences in postprandial responses between taurine- and glycine-conjugated bile acids. Bile acids that escape reabsorption can be deconjugated by bacteria with bile salt hydrolase activity and then further metabolized into secondary bile acids by dehydroxylation, dehydrogenation, or epimerization.^19^ Unconjugated and secondary bile acids are reabsorbed by active or passive transport in the small and large intestine.^39^

Both gastric bypass procedures in this study; RYGB and MGB, have the upper part of the duodenum bypassed, and subsequently, food and bile acids do not mix until they reach the common channel in the jejunum. This may lead to a faster passage of conjugated bile acids through the small intestine and potentially, increased passive absorption of bile acids in jejunum as well as active absorption in ileum, which could explain the earlier postprandial response of bile acids after surgery. Subsequently, microbial conversion of bile acids can also occur earlier after the meal than before surgery, which leads to a faster uptake and a detectable postprandial response of some unconjugated bile acids after surgery. Enhanced bile acid absorption could be related to reduced possibility of bile acids to form mixed micelles with fat, which might affect lipid absorption.

Most of the secondary bile acids such as HCA, HDCA, LCA, isoLCA, isoDCA isoUDCA and different oxo-bile acids did not show a postprandial response suggesting that they are more dependent on microbial metabolism and transit rather than release of primary bile acids from the gallbladder. Alternatively, there could be differences in expression of bile acid transporters after surgery and differences in structure and hydrophobicity of different bile acids can influence the uptake via passive diffusion.^18^^,^^39^^,^^40^ It has been demonstrated that unconjugated bile acids are absorbed more rapidly in ileum and jejunum than the corresponding glycine-conjugates due to higher permeability of free bile acids, and that the jejunal permeability of CDCA are three times higher than the permeability of CA.^41^

We observed that iso-bile acids: isoLCA, isoDCA and isoUDCA, from microbial metabolism decreased in plasma after surgery and the levels of iso-LCA also decreased in faeces. This could be due to changes in the gut microbiota composition and/or changes in the environment of the gut after surgery, which might influence bacterial transformation of bile acids. This is in agreement with a recent study, which showed that iso-UDCA levels were decreased after bariatric surgery and established that iso-UDCA levels were determined by the gut microbiota and could also be decreased after fiber supplementation.^42^ Another study showed that iso-LCA was associated with inhibition of T_H_17 cells and may be implicated in the pathophysiology of inflammatory disorders.^43^^,^^44^ Connections between bile acids and immune regulation have been further investigated and both FXR and TGR5 have demonstrated anti-inflammatory effects.^45^, ^46^, ^47^

It is important to emphasize that only some bile acids show alterations in the fasted state and alterations in other bile acids might be neglected if they are not analysed after a meal challenge. A study by Lalloyer et al. showed that total fasting plasma bile acid levels increased one year after RYGB, but they did not measure the postprandial response of bile acids or faecal bile acids.^48^ They analysed expression of hepatic genes involved in bile acid synthesis but did not observe any changes in the expression of *CYP7A1*, which is the rate-limiting enzyme in the classical pathway, or *CYP8B1*, which is responsible for CA formation. Instead, they attributed the increase in plasma bile acid levels to an upregulation of genes involved in the alternative bile acid synthetic pathway.^48^ However, our results indicate that the increased plasma bile acid levels after surgery are mainly caused by decreased faecal bile acid excretion and increased recirculation of bile acids. This argument is strengthened by the decreased fasting C4 levels after surgery, which was also found in the study by Lalloyer et al., indicating that bile acid synthesis is reduced after bariatric surgery.

We observed an increased postprandial response of FGF19 after surgery, especially in patients with T2D at surgery, which is in line with previous findings that FGF19 is reduced in patients with obesity and that the levels are restored after bariatric surgery.^28^^,^^49^ FGF19 is a direct target for the nuclear receptor FXR, and thus the findings indicate that there is increased FXR activation in the small intestine after surgery, especially in the patients with T2D at the time of surgery. This is consistent with the increased bile acid levels and reduced C4 levels and supports that FXR is essential for the positive effects on weight loss and metabolism after surgery.^25^^,^^26^

The 6α-hydroxylated bile acids, HDCA and GHCA, were increased in the fasted state after surgery in the BARIA cohort, consistent with previous findings.^48^^,^^50^ The levels of HDCA covaried with diabetes remission in the Oseberg study, suggesting that HDCA may contribute to diabetes remission, which is in line with a previous study where serum levels of HCA species could predict diabetes remission.^50^ Plasma levels of HDCA were relatively low, within a 10 nM range, and the physiological relevance of such low concentrations would need to be further investigated. However, previous studies by our group have shown that another microbially produced metabolite, imidazole propionate, has a large impact on host physiology even at similarly low concentrations.^51^

We have previously shown that mice fed oligofructose have increased circulating levels of 6α-hydroxylated bile acids, and that HDCA production was microbially dependent.^52^ Mechanistically, HDCA activates TGR5 on L-cells and promotes secretion of the incretin GLP-1.^52^^,^^53^ Interestingly, the glucose-lowering effects required intact GLP1R signalling. It is intriguing that a similar shift in bile acid metabolism, i.e., increased levels of 6α-hydroxylated bile acids and decreased levels of iso-bile acids, can be induced by either bariatric surgery or by fiber supplementation,^42^^,^^52^ and further studies to find common denominators behind the mechanisms are required.

In conclusion, we have identified changes in bile acid kinetics after bariatric surgery and showed that a mixed-meal challenge is required to observe changes in some bile acids, while others are altered in the fasted state. We provide evidence that the FXR/FGF19 pathway is activated following bariatric surgery of patients with obesity. In addition, we also demonstrate that fasting plasma levels of HDCA are increased after surgery and are associated with metabolic improvements and diabetes remission. The bile acid data obtained in this study is extensive and our aim was to present it in a clear and informative way, although we are aware that the data could have been analysed in different ways. We quantified the maximum rise in relation to the fasting value to demonstrate the magnitude of the postprandial response, and we determined the timepoint of maximum rise to shed light on changes in bile acid kinetics. By dichotomising the response into the three categories; early, late and no response, we could decipher the complex data and get an overall view of the different bile acids.

One limitation with our study is that only 16 patients were diagnosed with T2D before surgery and that a combination of two different surgery types were used in this group. Additional studies with larger cohorts including more patients with T2D will be essential to draw further conclusions on the metabolic effects of bile acids on T2D remission after bariatric surgery. Another limitation is that the measurement after surgery was not performed until after one year, thus earlier measurements would be required to draw conclusions on more acute changes in bile acid kinetics after bariatric surgery. It would also be important to study the postprandial response of bile acid kinetics after sleeve gastrectomy and compare the effect of the different surgical procedures.

## Contributors

Conceptualization: A.W., L.O., Ö.A., A.G., M.N. and F.B.; Clinical Investigation/Sample acquisition: Ö.A., R.F., A.v.d.L., A.S.M., V.G., M.N., D.H. and J.H.; Methodology: L.O., A.W., A.L., M.H. and W.S.; Formal analysis: A.W., L.O. and W.S.; Visualization: A.W. and L.O.; Verification of underlying data A.W. and L.O; Resources, M.N. and F.B.; Writing—Original Draft, A.W. and L.O.; Writing—Review & Editing: A.W., L.O., W.S., A.G., H-U.M., J.H., M.N. and F.B. All authors have read and approved the final version of the manuscript.

## Data sharing statement

Requests for deidentified data can be addressed from the corresponding author.

## Declaration of interests

F.B. receives research funding from Biogaia AB, is co-founder and shareholder of Roxbiosens Inc and Implexion AB, and is on the scientific advisory board of Bactolife A/S. L.O is co-founder and shareholder of Roxbiosens Inc. V.G. receives research funding from Spaarne Gastuis. M.N is member of the Scientific Advisory Board of and holds equity in Caelus Pharmaceuticals and receives research funding from and holds equity in Ami therapeutics, the Netherlands. However, none of these possible conflicts of interest bear direct relations to the content of the current paper.

## Appendix ASupplementary data

Supplementary Table S6

Supplementary Fig. S1 **Supplementary Fig. S1. Alterations in metabolic parameters after bariatric surgery.** (a and b) Plasma levels of glucose (a), insulin (b) at the different timepoints during the mixed-meal test before (PRE) and after (POST) surgery in patients without T2D (nonT2D) and with pre-surgery T2D (T2D). nonT2D group; green colors, T2D group; pink colors, PRE; light color, POST; dark color. ∗p < 0.05, ∗∗p < 0.01, indicate differences between nonT2D and T2D groups before or after surgery using Multiple Mann–Whitney tests. Mean ± SEM are shown in the graphs. (PRE: nonT2D glucose, n = 21; T2D glucose, n = 10; nonT2D insulin, n = 20, T2D insulin, n = 12; nonT2D; POST: nonT2D glucose, n = 11; T2D glucose, n = 10; nonT2D insulin, n = 11, T2D insulin, n = 11).
