# Supplementary figures and images for: Alterations in bile acid kinetics after bariatric surgery in patients with obesity with or without type 2 diabetes
Source: eBioMedicine. 2024 Aug 2;106:105265. doi: 10.1016/j.ebiom.2024.105265 (PMC11345581; doi:10.1016/j.ebiom.2024.105265)

Supplemental figure 1

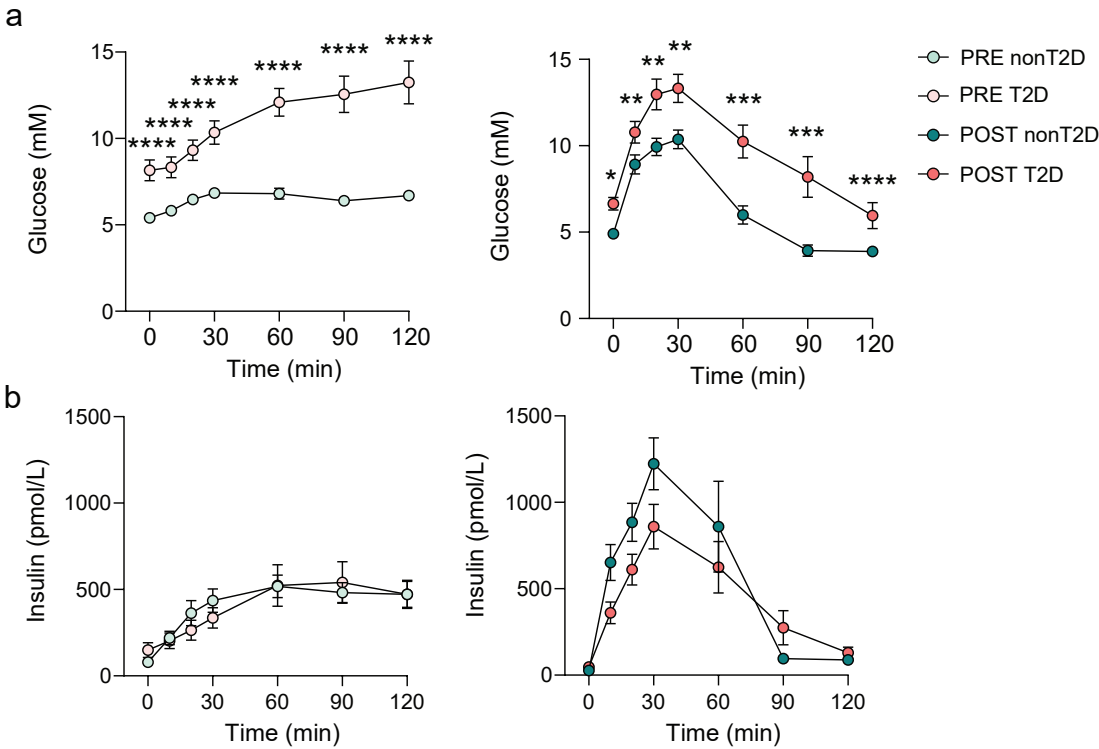

Supplement: Supplementary Fig. S1 — Alterations in metabolic parameters after bariatric surgery. (a and b) Plasma levels of glucose (a), insulin (b) at the different timepoints during the mixed-meal test before (PRE) and after (POST) surgery in patients without T2D (nonT2D) and with pre-surgery T2D (T2D). nonT2D group; green colors, T2D group; pink colors, PRE; light color, POST; dark color. ∗p < 0.05, ∗∗p < 0.01, indicate differences between nonT2D and T2D groups before or after surgery using Multiple Mann–Whitney tests. Mean ± SEM are shown in the graphs. (PRE: nonT2D glucose, n = 21; T2D glucose, n = 10; nonT2D insulin, n = 20, T2D insulin, n = 12; nonT2D; POST: nonT2D glucose, n = 11; T2D glucose, n = 10; nonT2D insulin, n = 11, T2D insulin, n = 11). [file mmc7.pdf]
